# Mechanical activities of self-beating cardiomyocyte aggregates under mechanical compression

**DOI:** 10.1038/s41598-021-93657-z

**Published:** 2021-07-26

**Authors:** Ken Nakano, Naoya Nanri, Yoshinari Tsukamoto, Mitsuru Akashi

**Affiliations:** 1 Yokohama National University, 79-7 Tokiwadai, Hodogaya, Yokohama, Kanagawa 240-8501 Japan; 2 Osaka University, 1-3 Yamadaoka, Suita, Osaka 565-0871 Japan

**Keywords:** Mechanical engineering, Biomaterials, Biological physics

## Abstract

Since the discovery of synchronous pulsations in cardiomyocytes (CMs), electrical communication between CMs has been emphasized; however, recent studies suggest the possibility of mechanical communication. Here, we demonstrate that spherical self-beating CM aggregates, termed cardiac spheroids (CSs), produce enhanced mechanical energy under mechanical compression and work cooperatively via mechanical communication. For single CSs between parallel plates, compression increased both beating frequency and beating energy. Contact mechanics revealed a scaling law on the beating energy, indicating that the most intensively stressed cells in the compressed CSs predominantly contributed to the performance of mechanical work against mechanical compression. For pairs of CSs between parallel plates, compression immediately caused synchronous beating with mechanical coupling. Compression tended to strengthen and stabilize the synchronous beating, although some irregularity and temporary arrest were observed. These results suggest that mechanical compression is an indispensable control parameter when evaluating the activities of CMs and their aggregates.

## Introduction

Although individual differences inevitably exist between two pendulum clocks, flexible common support may allow their oscillatory motions to interact, keeping their rhythms perfectly matched until one of them stops. This fascinating phenomenon, synchronization in coupled oscillators^1^, discovered by Christiaan Huygens in 1665^2^, is a universal phenomenon emerging in various fields, such as mechanical^2–6^, electrical^7,8^, chemical^9,10^, and biological^11,12^ systems. From a mechanical standpoint, what creates these cooperative phenomena is the variability of oscillation frequency and the interaction (in other words, communication) between oscillators. In physiology and medical science, the synchronization of cardiomyocytes (CMs) has attracted many researchers for many decades^13–16^ as a fundamental phenomenon of the heartbeats that sustain our lives.

Needless to say, the heart is a *biological* organ functioning as a *mechanical* pump^17^. A number of CMs in a heart contract synchronously, and the total mechanical work circulates blood throughout the body^17^. In an in vivo heart, CMs located at the sinoaterial node act as the primary pacemaker and govern the contraction of the other CMs via electrical communication^7,17^. Even isolated in vitro, CMs contract spontaneously and rhythmically at the same approximate frequency as in vivo hearts^18,19^. In addition, although a pair of CMs contract at their respective frequencies due to individual differences, direct contact with each other makes them synchronous within dozens of minutes^20^. The same is true for synchronization in a single cluster of CMs^18,21,22^, between two clusters^23^, in a heterocellular culture model^24^, and in a CM network model^25^; a number of direct contacts between CMs form electrical connections to enable them to communicate electrically^18–25^. Consequently, electrical communication between CMs is undoubtedly vital for their synchronization.

However, the existence of electrical communication does not preclude other types of communication. Considering that an isolated CM behaves like a self-excited *mechanical* oscillator^26^, there may exist another mechanism of mechanical communication that achieves the synchronous beating of CMs even with no electrical communication. Alternatively, there may exist a secondary mechanism by which mechanical coupling assists the synchronous beating of CMs established by electrical communication.

In recent years, the effects of mechanical stimulation on various types of cells have been investigated in the interdisciplinary field of cell mechanics^27^, and CMs are no exception. For example, the mechanical properties of a single CM have been measured using the deformations of AFM cantilevers^28–30^, elastic substrates^31,32^, and elastic micropillars fabricated on substrates^33–35^. In particular, the effect of substrate stiffness is remarkable: adequately flexible substrates stabilize beating CMs^32^, and substrate stiffness increases their twitch power^31,35^. Mechanical stimuli applied to substrates cause nonbeating CMs to begin activity^36^, meaning that CMs have an internal mechanism of sensing mechanical stimulation (i.e., mechanical–electrical feedback)^37–39^. In addition, recent reports on the forced entrainment of a single CM to substrate oscillations^40^ and the mutual entrainment of a pair of CMs via substrate deformation^36^ indicate the possibility of mechanical communication between CMs^36,40–42^. However, CMs intrinsically work mechanically under external load. With that in mind, it would be not unnatural to think that a different key to opening the door between in vitro and in vivo CMs may lie in the mechanical work done by CMs under mechanical load.

**Figure 1:**
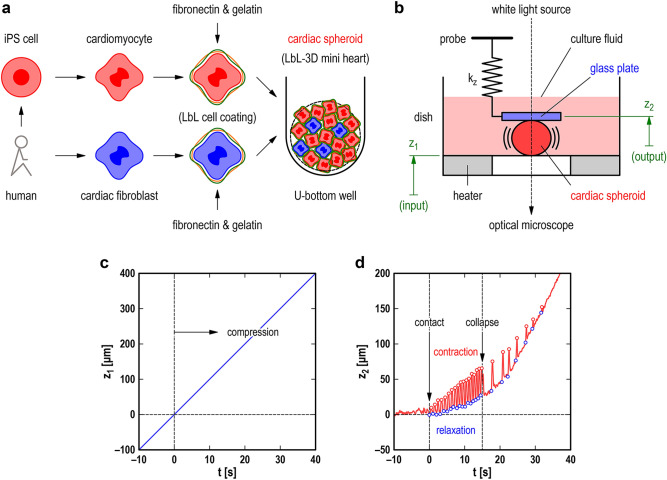
The mechanical response of a single cardiac spheroid (CS) to increasing compression. (**a**) A CS was fabricated by assembling human induced pluripotent stem cell-derived cardiomyocytes and human cardiac fibroblasts in a U-bottom well via layer-by-layer cell coating of fibronectin and gelatin. (**b**) A transparent polystyrene dish in which a CS was immersed in a culture fluid at a controlled temperature of 37 °C by a heater was lifted upward at a predetermined constant speed *V*_*z*_ (= d*z*_1_/d*t*). At *t* = 0, the CS made contact with two horizontal surfaces: the dish surface and the glass plate surface, where the glass plate (diameter: 1.0 mm and thickness: 0.2 mm) was mounted at the tip of a flexible probe with vertical stiffness *k*_*z*_. The compression of the probe spring was measured under an optical microscope. (**c**) Temporal change in the vertical position of the lower surface, *z*_1_ (as the input into the system). (**d**) Temporal change in the vertical position of the upper surface, *z*_2_ (as the output from the system). The blue and red circles denote the local minimum (i.e., CS relaxation) and local maximum (i.e., CS contraction), respectively. Experimental conditions: *D* = 340 μm (*N*_cell_ ~ 8000), *k*_*z*_ = 3.4 N/m, and *V*_*z*_ = 10 μm/s.

Here, we demonstrate that through *macroscopic* experimental investigations based on contact mechanics^43–45^, *tiny* self-beating CM aggregates of spherical shape, termed cardiac spheroids (CSs), produce enhanced mechanical energy under mechanical compression and work cooperatively via mechanical communication. The CSs were human derived, hundreds of micrometers in diameter, fabricated by assembling 75% human induced pluripotent stem cell-derived CMs (hiPSC-CMs) and 25% human cardiac fibroblasts (see Fig. 1a). Note hiPSC-CMs are generally considered immature and exhibit spontaneous beating^46,47^. A single CS or a pair of CSs were placed between a pair of horizontal surfaces (see Fig. 1b); the lower surface was the top surface of a dish containing culture fluid, and the upper surface was the bottom surface of a glass plate mounted at the tip of a vertical probe spring. During compression of the CS(s) between the two surfaces, the spring deflection was measured under an optical microscope. Using a spring as flexible as the tested CSs enabled us to apply a moderate load and acquire their static and dynamic mechanical responses. The spring deflection calculated the potential energy (i.e., the sum of the elastic strain energy and gravitational energy) of the probe, enabling us to quantify the mechanical work done by the CSs directly. In addition, in the tests on a pair of CSs, the probe acted as a mechanical coupling for their mechanical communication.

## Results

In this section, we describe the mechanical response of human-derived self-beating CSs (Fig. 1a) observed in two types of tests: (i) compression tests on single CSs and (ii) compression tests on pairs of CSs, where a homemade apparatus (Fig. 1b) was used for both types of tests. A single CS or a pair of CSs was placed between the two parallel surfaces and gradually compressed by lifting the lower surface upward at a constant speed *V*_*z*_. In both types of tests, as a function of time *t*, the input signal was the vertical position of the lower surface, *z*_1_ = *z*_1_(*t*), while the output signal was the vertical position of the upper surface, *z*_2_ = *z*_2_(*t*), which was mounted at the tip of the probe spring with the vertical stiffness *k*_*z*_.

### Compression tests on single CSs

A single CS with a diameter *D* = 340 μm (the total number of cells *N*_cell_ ~ 8000) was tested with a probe of *k*_*z*_ = 3.4 N/m at the drive speed *V*_*z*_ = d*z*_1_/d*t* = 10 μm/s (Fig. 1c), where the origin of *z*_1_ was determined so that *z*_1_ = 0 at *t* = 0. The temporal change in *z*_2_ (Fig. 1d), with the help of optical microscope observation, determined the starting time of firm contact, *t* = 0, between the CS and the two surfaces. The origin of *z*_2_ was determined so that *z*_2_ = 0 for *t* < 0, where the buoyancy effect and electrical high-frequency noise were removed from the measured signal. For *t* > 0, the CS was gradually compressed, and the static compression and dynamic beating produced the response *z*_2_. The local minimum values, represented by blue circles, were due to “CS relaxation”, while the local maximum values, represented by red circles, were due to “CS contraction”. We found that increasing the compression caused faster and stronger CS beating until excessive compression collapsed the CS at *t* ~ 15 s. Similar behaviors from contact to collapse were observed for CSs of different sizes (e.g., *N*_cell_ ~ 2000 or 4000) at different compression rates (e.g., *V*_z_ = 5 μm/s). As a result, the critical parameter in the compression tests was the probe stiffness: if it was inappropriately hard (e.g., *k*_*z*_ = 28.4 N/m), it was difficult to detect the dynamic beating.

**Figure 2:**
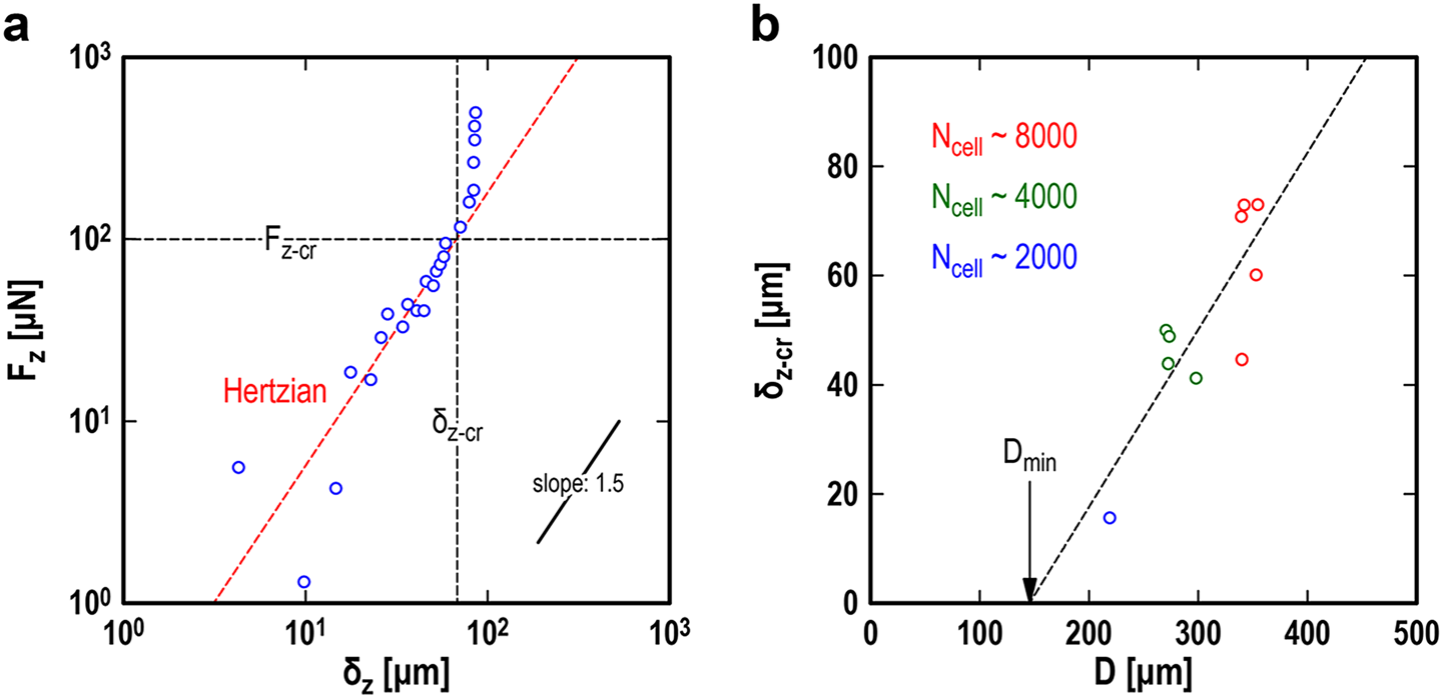
The static properties of single cardiac spheroids (CSs). (**a**) The normal load, *F*_*z*_ = *k*_*z*_*z*_2_, is plotted against the indentation depth, *δ*_*z*_ = (*z*_1_–*z*_2_)/2, for the local minimum values of *z*_2_(*t*) (i.e., the blue circles in Fig. 1d). The red broken line is a fitting function with a slope of 1.5 based on Hertzian contact theory for the contact between a sphere and a plane, used to evaluate the elasticity of the tested CS (e.g., its effective elastic modulus is *E* = 5.2 kPa). (**b**) The critical indentation depth *δ*_*z*-cr_ at which the CS was collapsed by compressive loading is plotted against the CS diameter, *D*, and is used to evaluate the toughness of the tested CSs. The black broken line is a linear fitting function intersecting the horizontal axis at *D* = *D*_min_ = 146 μm. The plotted data in (**b**) are for ten different CSs, the diameters of which are ranged from 219 to 355 μm.

The “CS relaxation” data provide two types of static properties: the elasticity (Fig. 2a) and toughness (Fig. 2b) of CSs. Figure 2a is a double-logarithmic plot of the normal load *F*_*z*_ = *k*_*z*_*z*_2_ against the indentation depth *δ*_*z*_ = (*z*_1_–*z*_2_)/2, where *z*_1_- and *z*_2_-values are provided from those that appeared at relaxation (i.e., blue circles in Fig. 1d). We found that the plotted data for *δ*_*z*_ < *δ*_*z*-cr_ follow the relationship *F*_*z*_ ~ *δ*_*z*_^1.5^, as predicted by Hertzian contact theory^43–45^ for the contact between a sphere and a plane, where *δ*_*z*-cr_ is the critical indentation depth at which the CS collapsed due to compression. This means that the CS in relaxation underwent macroscopically elastic deformation, even though it was tiny and consisted of nonuniform cells. The fitting function, represented by the red broken line, provides an effective Young’s modulus *E* = 5.2 kPa. Figure 2b is a plot of *δ*_*z*-cr_-values against *D*-values obtained similarly for ten different CSs. The linear fitting function, represented by the black broken line, shows a clear positive correlation between the two quantities and intersects with the horizontal axis at *D* = *D*_min_ > 0. This indicates that the diameter of a CS fabricated by this method cannot be smaller than *D*_min_; otherwise, an infinitesimal load would collapse it.

**Figure 3:**
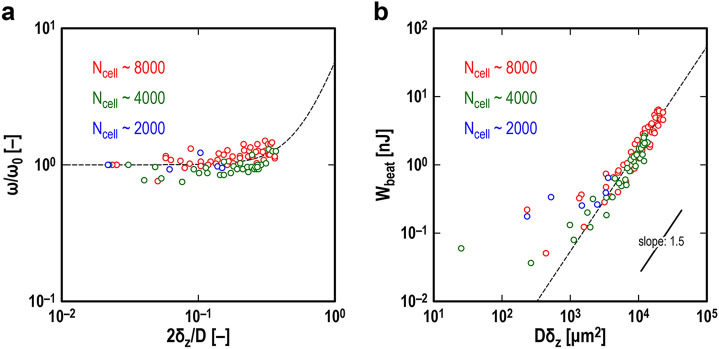
The dynamic properties of single cardiac spheroids (CSs). (**a**) The normalized beating frequency, *ω*/*ω*_0_, is plotted against the normalized indentation depth (i.e., effective compression strain) 2*δ*_*z*_/*D*, where *ω* and *ω*_0_ are the beating frequencies under compression and no compression, respectively. The black broken line is a fitting function of *ω*/*ω*_0_ = 1 + *a* (2*δ*_*z*_/*D*)^*n*^, where the fitting parameters were determined to be *a* = 4.7 and *n* = 2.8 by least square analysis. (**b**) The mechanical work performed by a single CS in a single contraction, termed beating energy *W*_beat_, is plotted against *Dδ*_*z*_. The black broken line is a fitting function of *W*_beat_ = *b* (*Dδ*_*z*_)^1.5^, where the fitting parameter was determined to be *b* = 1.7 × 10^–6^ nJ/μm^3^ by least square analysis. The plotted data in (**a**) and (**b**) are from 94 contractions of six different CSs, the diameters of which are ranged from 219 to 355 μm.

On the other hand, using the “CS contraction” data clarifies two types of dynamic properties: the beating frequency (Fig. 3a) and beating energy (Fig. 3b) of CSs. Figure 3a is a double-logarithmic plot of the normalized beating frequency *ω*/*ω*_0_ against the normalized indentation depth (in other words, effective compression strain) 2*δ*_*z*_/*D*, where *ω* is the instantaneous beating frequency obtained from a time interval between adjacent contractions, and *ω*_0_ is the beating frequency under no compression. The *δ*_*z*_-value for the horizontal axis was provided from the value at relaxation between the two contractions. The plotted data obtained from 94 contractions of six different CSs appear to be approximated well by an empirical fitting function *ω*/*ω*_0_ = 1 + *a* (2*δ*_*z*_/*D*)^*n*^: small compression has little effect on beating frequency, while larger compression (e.g., 2*δ*_*z*_/*D* > 0.1) tends to make the beating faster. The coefficient value *a* = 4.7 determined by least square analysis indicates that the increase in beating frequency due to compression is up to several fold. Figure 3b is a double-logarithmic plot of the beating energy *W*_beat_ against the product of the CS diameter *D* and indentation depth *δ*_*z*_. Note that the beating energy *W*_beat_ is the mechanical work that a single CS performed in a single contraction: by using the potential energy of the probe, *U* = *k*_*z*_*z*_2_^2^/2, it was quantified as the difference between *U* at the contraction and *U* at the immediately preceding relaxation. The *δ*_*z*_-value for the horizontal axis was that at relaxation. Surprisingly, most of the data points are located close to the fitting function with a slope of 1.5 except for several data points with small *W*_beat_ values. This means that there exists a strong law regarding CS beating energy due to compression: *W*_beat_ ~ (*Dδ*_*z*_)^1.5^. The meaning of this scaling law will be discussed later in detail.

### Compression tests on pairs of CSs

**Figure 4:**
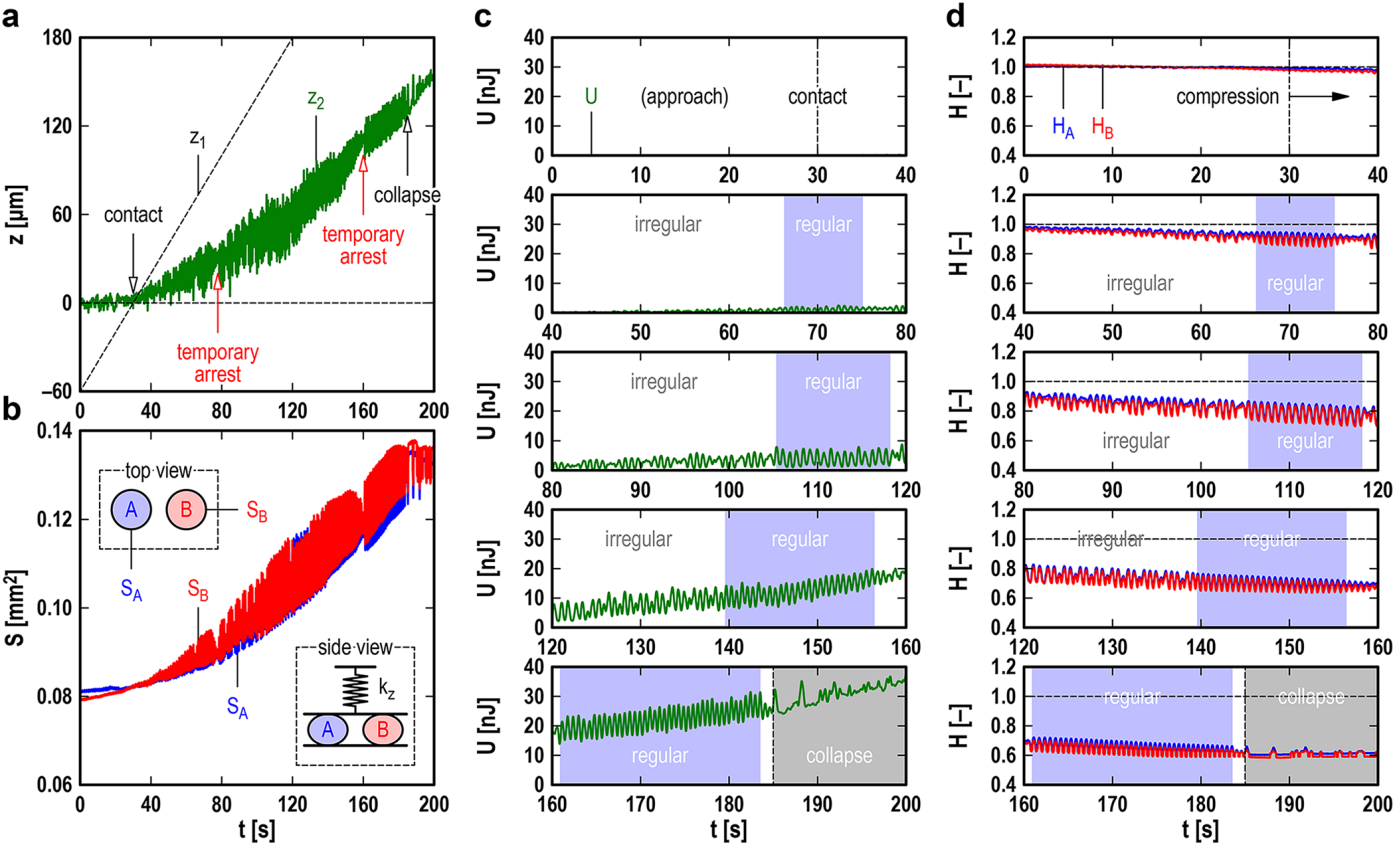
The mechanical response of a pair of cardiac spheroids (CSs) to increasing compression. (**a**) Temporal changes in the vertical position of the lower surface, *z*_1_ (diagonal broken line), and the vertical position of the upper surface, *z*_2_ (green line). (**b**) Temporal changes in the projected areas of the CSs, *S*_A_ (blue line) and *S*_B_ (red line), observed by optical microscopy. (**c**) Temporal change in the potential energy of the probe, *U* = *k*_*z*_*z*_2_^2^/2 (green line). (**d**) Temporal changes in the normalized heights of the CSs, *H*_A_ = *S*_A0_/*S*_A_ (blue line) and *H*_B_ = *S*_B0_/*S*_B_ (red line), where *S*_A0_ and *S*_B0_ are the projected areas of the CSs under no compression. The light blue bands in (**c**) and (**d**) represent the periods of regular synchronous beating. Experimental conditions: *D*_A_ = 322 μm (*N*_cell-A_ ~ 4000), *D*_B_ = 320 μm (*N*_cell-B_ ~ 4000), *k*_*z*_ = 3.4 N/m, and *V*_*z*_ = 2 μm/s.

A pair of CSs, termed CS-A (*D*_A_ = 322 μm and *N*_cell-A_ ~ 4000) and CS-B (*D*_B_ = 320 μm and *N*_cell-B_ ~ 4000), were placed between the two surfaces of the apparatus and tested in the same manner as a single CS, with a probe of *k*_*z*_ = 3.4 N/m at the drive speed *V*_*z*_ = 2 μm/s. The CSs were initially set approximately 0.5 mm apart on the lower surface, and optical microscope observation confirmed that they did not come into direct contact with each other throughout the compression test. Figure 4a shows temporal changes in *z*_1_ (diagonal broken line) and *z*_2_ (green line). The CSs made firm contact with the two surfaces at *t* ~ 30 s, then underwent increasing compression, and finally collapsed at *t* ~ 185 s. Note that the starting time of contact, *t* ~ 30 s, is approximate because the sizes of the tested CSs were not exactly the same due to *inevitable* individual differences. Figure 4b shows temporal changes in the projected areas of the CSs: *S*_A_ (blue line) and *S*_B_ (red line) for CS-A and CS-B, respectively, were obtained by analyzing the optical images captured under the optical microscope (see the inset “top view” in Fig. 4b). As a result, it was found that they had enough resolutions to investigate the mechanical activities of the two CSs independently. Consequently, using the data in Fig. 4a provides a temporal change in the potential energy of the probe: *U* = *k*_*z*_*z*_2_^2^/2 (Fig. 4c). On the other hand, under the assumption that the CSs were deformed from spheres to ellipsoids while preserving their volumes, using the data in Fig. 4b provides temporal changes in the normalized heights of the CSs: *H*_A_ = *S*_A0_/*S*_A_ and *H*_B_ = *S*_B0_/*S*_B_ for CS-A and CS-B, respectively, where *S*_A0_ and *S*_B0_ are *S*_A_ and *S*_B_ under no compression, respectively (Fig. 4d).

Next, we take a closer look at Fig. 4c and d. First, under no compression (*t* < 30 s), the two CSs beat weakly and independently without interfering with each other. Magnified views of *U*, *H*_A_, and *H*_B_ at approximately *t* = 20 s are shown in Fig. S1 with snapshots from microscope observation in the Supplementary Information. The beating frequency under no compression was *ω*_A0_ = 0.86 Hz for CS-A and *ω*_B0_ = 1.54 Hz for CS-B, which caused the phase of CS-B to overtake that of CS-A repeatedly. However, immediately after the upper surface made firm contact with the two CSs at *t* ~ 30 s, no phase overtaking was observed, and the pair of CSs beat synchronously until they collapsed. Note that the positional relationship between the two CSs and the two surfaces was shown by the inset “side view” in Fig. 4b: the two CSs had no direct contact with each other and were allowed to communicate only mechanically via the probe consisting of the upper surface and the spring. Therefore, it is probable that the CS synchronization recognized in this test was achieved using only mechanical communication without electrical communication. The synchronous beating could be divided into two types: “regular” and “irregular” synchronous beating, where the light blue bands in Fig. 4c and d show the periods during which the former occurred. Magnified views of the second period of regular synchronous beating at approximately *t* = 110 s are shown in Fig. S2 similarly to Fig. S1 in the Supplementary Information. We found that the two CSs cooperatively produced considerable beating energy (e.g., *W*_beat_ ~ 5 nJ) through regular synchronous beating. Comparison between different periods showed that regular synchronous beating became longer and stronger with increasing compression.

**Figure 5:**
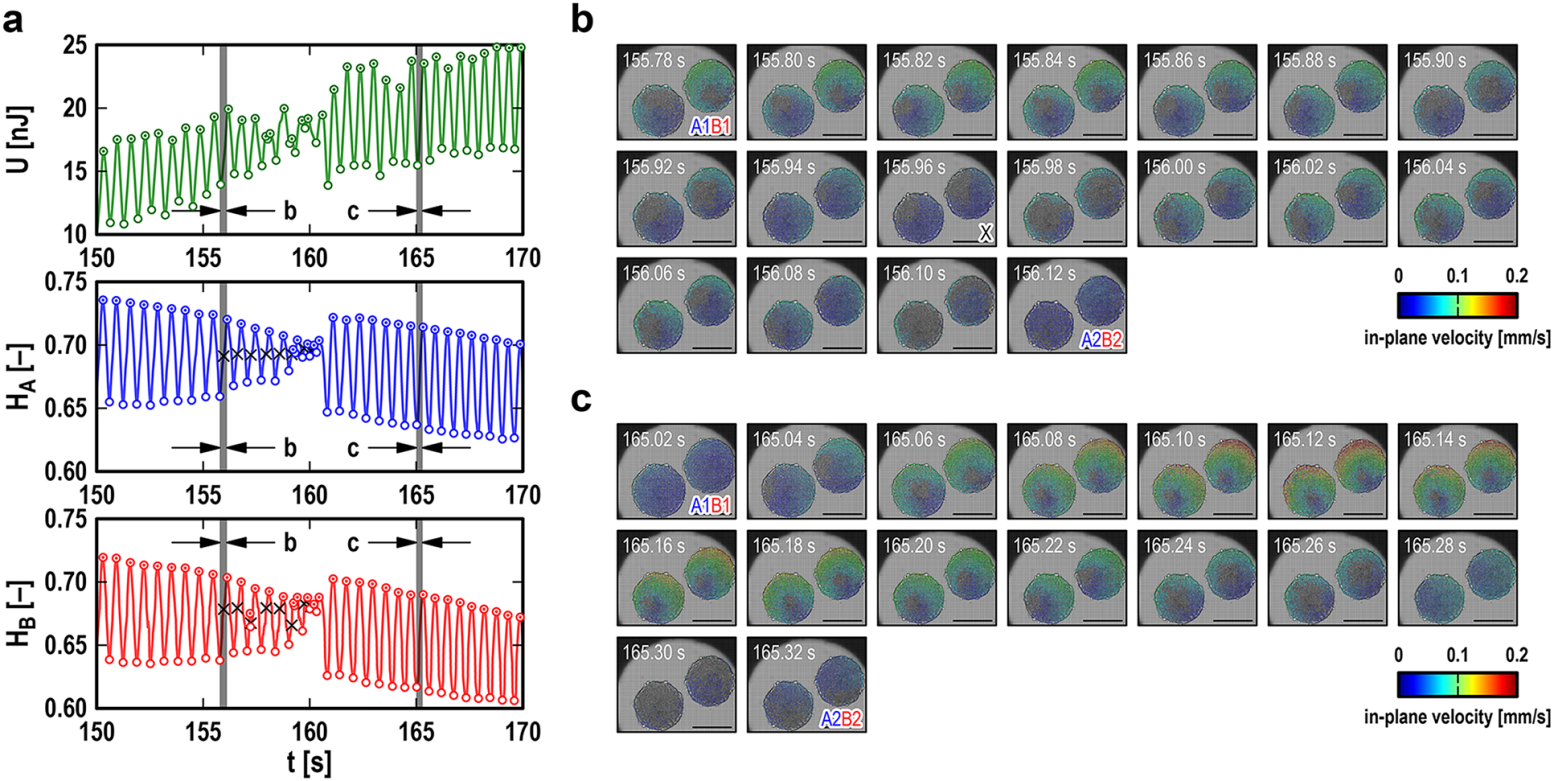
The temporary arrest of synchronous beating and its precursors in a pair of cardiac spheroids (CSs) under compression. (**a**) Temporal changes in the potential energy of the probe, *U* (top graph), and the normalized heights of the CSs, *H*_A_ (middle graph; for the left CS in the snapshots in **b** and **c**) and *H*_B_ (bottom graph; for the right CS in the snapshots in **b** and **c**), in the range of *t* = 150–170 s (from Figs. 4c and 4d). The open and dotted circles represent the local minimum (i.e., CS pair relaxation) and local maximum (i.e., CS pair contraction), respectively. The cross symbols represent the precursors of the *temporary arrest* (at *t* ~ 160 s) of synchronous beating. (**b**) Snapshots of the CSs in the range of *t* = 155.78–156.12 s. **c**: Snapshots of the CSs in the range of *t* = 165.02–165.32 s. The colors of the CSs in (**b**) and (**c**) represent the local in-plane velocity of the CSs calculated by particle image velocimetry. A1 and B1 are snapshots at the local minima of *H*_A_ and *H*_B_, respectively, and A2 and B2 represent the local maxima of *H*_A_ and *H*_B_, respectively. The snapshot at *t* = 155.96 s marked by X in (**b**) is an example of the precursors (i.e., the *momentary arrest* of the CS pair for less than 40 ms). The scale bar in each snapshot represents 0.3 mm.

In contrast to regular synchronous beating, several types of irregular synchronous beating were observed. For example, before the first regular period (e.g., *t* < 60 s), random oscillations of small amplitude appeared in *H*_A_ and *H*_B_. Irregular synchronous beating that appeared before the second and third regular periods might have been a set of several regular oscillations and short disturbed oscillations in between. Looking more carefully, we found a distinct “temporary arrest” of the measured system twice in the measured period from contact (*t* ~ 30 s) to collapse (*t* ~ 185 s). The first temporary arrest appeared at *t* ~ 80 s and the second at *t* ~ 160 s: both can be recognized as distinct “chips” in the coarse-grained original signals (see Fig. 4a and b). Figure 5a shows magnified views of *U*, *H*_A_, and *H*_B_ around the second temporary arrest. Before the temporary arrest, gradual weakening of the regular synchronous beating was observed (*t* < 156 s), followed by significant disturbances in *U* and *H*_B_ (*t* ~ 156–159 s). Immediately after the temporary arrest, regular synchronous beating appeared (*t* > 161 s) and produced considerable beating energy (*W*_beat_ ~ 8 nJ). Figure 5b and c show a series of snapshots of a single contraction process of the CS pair before and after the temporary arrest, respectively, from the local minimum (A1 and B1) to the local maximum (A2 and B2) of *H*_A_ and *H*_B_. The left and right CSs in the snapshots are CS-A and CS-B, respectively, and the color represents the distribution of in-plane velocity calculated by particle image velocimetry. In Fig. 5c, the in-plane velocity increases immediately after the local minimum (*t* = 165.02 s) and then decreases monotonically toward the local maximum (*t* = 165.32 s). However, in Fig. 5b, an unnatural “momentary arrest” of the CS pair was observed at *t* = 155.96 s in the process of reducing the in-plane velocity. The interval of the snapshots suggests that the duration of momentary arrest was less than 40 ms. All snapshots were examined, and *H*_A_ and *H*_B_ at the time of momentary arrest are marked by cross symbols in Fig. 5a. Since the momentary arrest of the CS pair is obviously localized a few seconds before the temporary arrest of synchronous beating, it is natural to believe that momentary arrest was a precursor to temporary arrest.

Compression tests performed for different pairs of CSs (e.g., diameters: *D*_A_ = 314 μm and *D*_B_ = 321 μm; beating frequency under no compression: *ω*_A0_ = 0.73 Hz and *ω*_B0_ = 0.68 Hz) showed results qualitatively similar to those described above. Thus, we can say that the phenomena shown in Figs. 4, 5, S1, and S2 for the specific CS pair (i.e., the independent beating of CSs under no compression, regular and irregular synchronous beating under compression, the temporary arrest of synchronous beating, and the momentary arrest of CSs as a precursor to the temporary arrest) have some generality.

## Discussion

Our compression tests between two parallel surfaces revealed the fundamental properties of self-beating *biological* CSs as self-excited *mechanical* oscillators^26^. An important aspect is that under mechanical compression, the CS acts as a mechanical oscillator that contracts periodically to recover its deformation. As a result of this contraction, the CS performs finite mechanical work. However, the amount of recovery due to contraction does not exceed the amount of deformation before contraction. For example, as shown in Fig. 4d, the normalized heights of CSs (i.e., *H*_A_ and *H*_B_) are always less than unity even during contraction. This means that self-beating CSs do not stretch themselves spontaneously: the mechanical compression applied to them could be a trigger for their strong mechanical beating.

Another important aspect is that the *dynamic* beating energy that a single CS produces in a single contraction strongly depends on the *static* mechanical compression applied immediately before the contraction. Immature CMs such as hiPSC-CMs beat spontaneously alone^18,19,46,47^; aggregates such as CSs consisting of immature CMs, sometimes termed “mini heart tissue”^48^, also beat spontaneously as a whole^18,21,22^, utilizing the mechanical–electrical feedback of CMs^37–39^ and the electrical communication between CMs in direct contact^18–25^. However, in reality, the mechanical pulsations of CSs were extremely weak unless they were compressed mechanically (Fig. S1). Therefore, the critical question is as follows: What is the mechanism of determining the beating energy of CSs? We found experimentally that the scaling law *W*_beat_ ~ (*Dδ*_*z*_)^1.5^ applied to the beating energy of single CSs (Fig. 3b), where *D* is the CS diameter under no compression, and *δ*_*z*_ is the penetration depth due to the mechanical compression applied immediately before mechanical pulsation. On the other hand, according to the qualitative evaluation method developed in contact mechanics^44^, letting *L*_c_ be the characteristic length of the contact area, we can estimate the volume where the compressive deformation is concentrated as *V*_c_ ~ *L*_c_^3^. In addition, according to Hertzian contact theory^43–45^, the radius of the circular contact area between a sphere and a plane is given by *a*_H_ = (*Dδ*_*z*_)^0.5^. Naturally, taking *L*_c_ = 2*a*_H_, we immediately find *V*_c_ ~ (*Dδ*_*z*_)^1.5^, leading to the relationship *W*_beat_ ~ *V*_c_. Thus, we can conclude that of the thousands of cells that make up a single CS, cells that are stressed by mechanical compression effectively perform mechanical work (conversely, unstressed cells rarely work). To be clear, the hypothesis was that if all cells in a CS worked, then *W*_beat_ ~ *D*^3^, or if the cells on the surface mainly worked, then *W*_beat_ ~ *D*^2^, though both assumptions were wrong.

The other important aspect is that the beating frequency of CSs is also affected by mechanical compression (Fig. 3a): obviously, variability in beating frequency is one of the requirements for synchronization^1^. For example, a pair of metronomes placed on a *horizontally movable common table* can overcome their individual differences to achieve synchronization^3,4^. Their frequency variation arises from the pendulum characteristics of the nonlinear soft spring, i.e., the frequency decreases as the amplitude increases^3,4^. In our compression tests on pairs of CSs, another requirement for synchronization seemed to be satisfied by a *vertically movable common surface*. This mechanical communication^36,40–42^ overcame the individual differences between the CSs (Fig. S1) and resulted in synchronous beating (Fig. S2). This synchronous beating of CSs appeared similar to the synchronous oscillations of metronomes^3^. However, in reality, that of CSs was much more complicated, showing regular and irregular beating alternating as compression increased (Fig. 4). The most prominent feature was the temporary arrest of synchronous beating (Fig. 5), reminding us of the amplitude death of coupled oscillators^49–51^ and sinus arrest of in vivo hearts^52–54^. Its mechanism seemed to be hidden in the momentary arrests of the CSs that appeared as its precursors. For example, in Fig. 5a, the *H*_A_ values for the precursors are all approximately 0.7, while the *H*_B_ values are scattered, suggesting that CS-A might have triggered the temporary arrest. In addition, before the temporary arrest, the local minimum of *H*_A_ increased from 0.65 (i.e., 35% compression) to 0.70 (i.e., 30% compression), suggesting that CS-A was hardened, possibly by some mechanical stimulus from CS-B with an inconvenient phase difference. This hardening of the CSs could be due to the internal structure and function of the cells^55,56^; the details remain to be clarified.

In summary, human-derived self-beating CSs work mechanically *under* and *against* mechanical compression. The frequency and amplitude of the CSs are functions of mechanical compression. When a single CS receives static compressive deformation, it contracts dynamically to recover the deformation according to the scaling law found in this study. For mechanically coupled CSs between parallel plates, they contract synchronously to recover the deformation cooperatively. However, this synchronous beating does not seem permanent and is not always stable. Although it is probably true that mechanical communication between CSs tries to make them synchronous, it also seems true that electrical communication is indispensable for the permanent and stable synchronous beating^18–25^. For future studies, it would be important to investigate the activities of CMs and their aggregates while controlling mechanical compression as a control parameter. In addition, considering regenerative medicine using CM sheets^57,58^, it would be meaningful to investigate the influences of other types of mechanical deformation, i.e., tensile and shear deformation. Hopefully, further studies would make it possible to optimize the CM layout in a CM aggregate to maximize its power and efficiency.

## Methods

### Fabrication of CSs

hiPSC-CM differentiation was based on our previous report^59^. hiPSC-CMs on day 13 of differentiation were used. Normal human cardiac fibroblasts (NHCFs, Lonza, Switzerland) were cultured in fibroblast growth medium (FGM-3, Lonza, Switzerland) at 37 °C in an incubator at 5% CO_2_. Extracellular matrix (ECM) nanofilms of fibronectin (FN) and gelatin (G) were formed on the surface of the cells using the filtration layer-by-layer (LbL) technique, according to our previous report^60^. For the fabrication of ECM nanofilms, a shaker (MixMate, Eppendorf, Germany) was used for the filtration LbL coating step. First, isolated cells were placed in a 6-well culture insert with a 3 μm pore polycarbonate membrane. The insert was immersed in phosphate-buffered saline (PBS) and agitated at 500 rpm for 1 min. Next, the cells washed with PBS were immersed in 0.2 mg/mL FN in PBS solution and agitated at 500 rpm for 1 min. Then, the FN-coated cells were immersed in PBS and agitated at 500 rpm for 1 min. After washing with PBS, the cells were immersed in 0.2 mg/mL G in PBS and agitated at 500 rpm for 1 min. With these steps as one cycle, four cycles were repeated. After the coating step cycles, the cells were immersed in FN solution and agitated at 500 rpm for 1 min. Finally, the cells coated with FN-G nanofilm were collected by Dulbecco's modified Eagle medium. The LbL-coated cells were mixed (LbL-hiPSC-CMs:LbL-NHCFs = 75:25) and seeded in a well plate (PrimeSurface MS-9096U, Sumitomo Bakelite, Japan) at 2000, 4000, and 8000 cells/200 μL. The seeded cells were incubated at 37 °C in an incubator at 5% CO_2_ to fabricate the CSs.

### Apparatus for compression tests

For the compression tests of the CSs, a homemade apparatus was used (see Fig. 1b), configured on the sample stage of an inverted optical microscope (AE2000, Shimadzu, Japan). The apparatus consisted of three types of units: a dish unit (Unit 1), a probe unit (Unit 2), and a sensor unit (Unit 3).

Unit 1: The dish unit consisted of a polystyrene dish (diameter: 40 mm, depth: 13 mm, Iwaki, Japan), a Peltier-type heater (TDC-1010A, Cell System, Japan), and 3D positioning stages. The vertical positioning stage was a motorized stage (KHE06008-CF, Suruga Seiki, Japan) to displace the dish and heater at a constant speed *V*_*z*_ = d*z*_1_/d*t* (see Fig. 1c) with a controller (DS102, Suruga Seiki, Japan). Unit 2: The probe unit consisted of a probe and 3D positioning stages. The probe was homemade, consisting of a V-shaped cantilever spring and a glass plate. The cantilever spring was made of a stainless steel sheet with a thickness of 0.1 mm for a stiffness of *k*_*z*_ = 3.4 N/m; the glass plate was made of borosilicate glass (D263, Schott, Germany) with a diameter of 1.0 mm and a thickness of 0.2 mm and was mounted at the tip of the cantilever spring. Unit 3: The sensor unit consisted of a laser-type displacement sensor (LK-H085, Keyence, Japan) and 3D positioning stages. The displacement sensor measured the cantilever spring deflection to calculate the vertical position *z*_2_ (see Fig. 1d) of the glass plate.

The electrical signal from the displacement sensor was recorded by a digital data logger (DL750, Yokogawa, Japan) at a sampling frequency of 50 Hz. The recorded signals were analyzed using MATLAB (MathWorks, USA). The optical images were captured at a frame rate of 50 frames per second by a digital camera (FL3-U3-13Y3M, FLIR Systems, USA) installed on the optical microscope. The captured images were analyzed using MATLAB and Flow-PIV (Library, Japan) to calculate the projected areas *S*_A_ and *S*_B_ (see Fig. 4b) and in-plane velocity distribution (see Fig. 5b) of CSs, respectively.

## Supplementary Information

Supplementary Information.
