# Supplementary material for: Mechanical activities of self-beating cardiomyocyte aggregates under mechanical compression
Source: Sci Rep. 2021 Jul 26;11:15159. doi: 10.1038/s41598-021-93657-z (PMC8313529; doi:10.1038/s41598-021-93657-z)
Supplement: Supplementary file 1 — Supplementary Information. [file 41598_2021_93657_MOESM1_ESM.pdf]

Supplementary information:

Mechanical activities of self-beating cardiomyocyte aggregates under mechanical compression

Ken Nakano[1,*], Naoya Nanri[1], Yoshinari Tsukamoto[2] & Mitsuru Akashi[2]

[1]Yokohama National University, 79-7 Tokiwadai, Hodogaya, Yokohama, Kanagawa 240-8501, Japan
[2]Osaka University, 1-3 Yamadaoka, Suita, Osaka 565-0871, Japan
[*]Corresponding author (email: nakano@ynu.ac.jp)

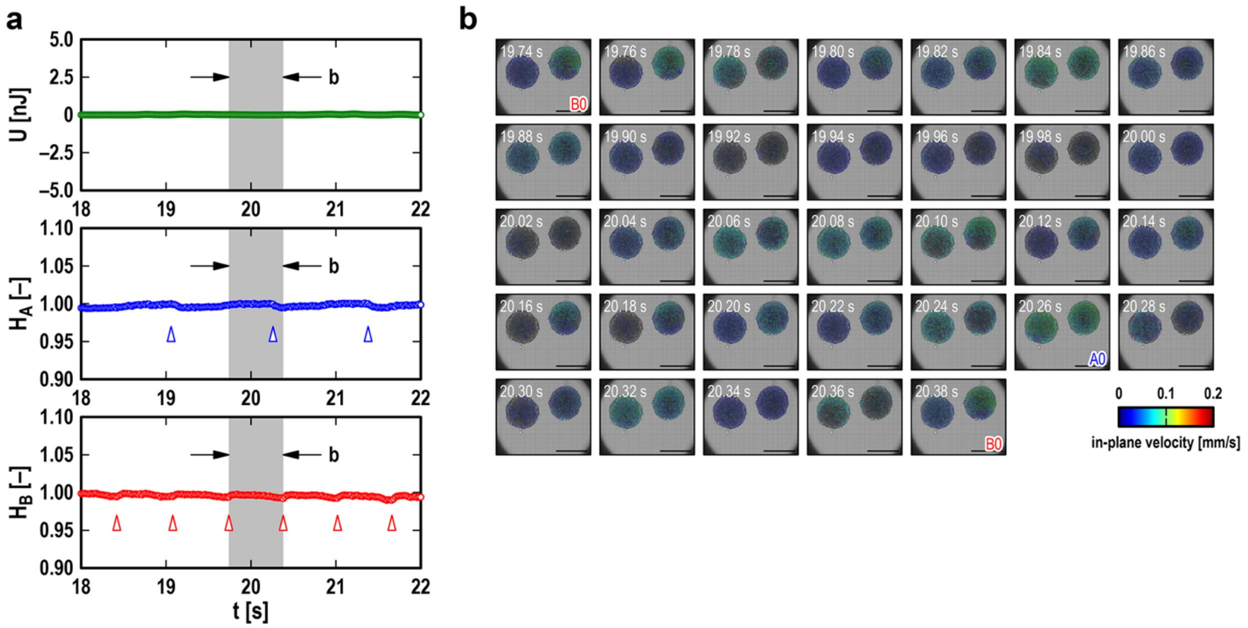

Figure S1 | The independent beating of a pair of cardiac spheroids (CSs) under no compression. a: Temporal changes in the potential energy of the probe, U (top graph), and the normalized heights of the CSs, H_A (middle graph; for the left CS in the snapshots in **b** and **c**) and H_B (bottom graph; for the right CS in the snapshots in **b** and **c**), in the range of t = 18–22 s (from **Figs. 4c** and **4d**). Blue and red triangles represent the beginning of the pulsive motion of CS A and B, respectively, denoted as A0 and B0 in **b**. **b**: Snapshots of the CSs in the range of t = 19.74–20.44 s (see the light gray band in **a**). The colors on the CSs represent the local in-plane velocity of the CSs calculated by particle image velocimetry. The scale bar in each snapshot represents 0.3 mm.

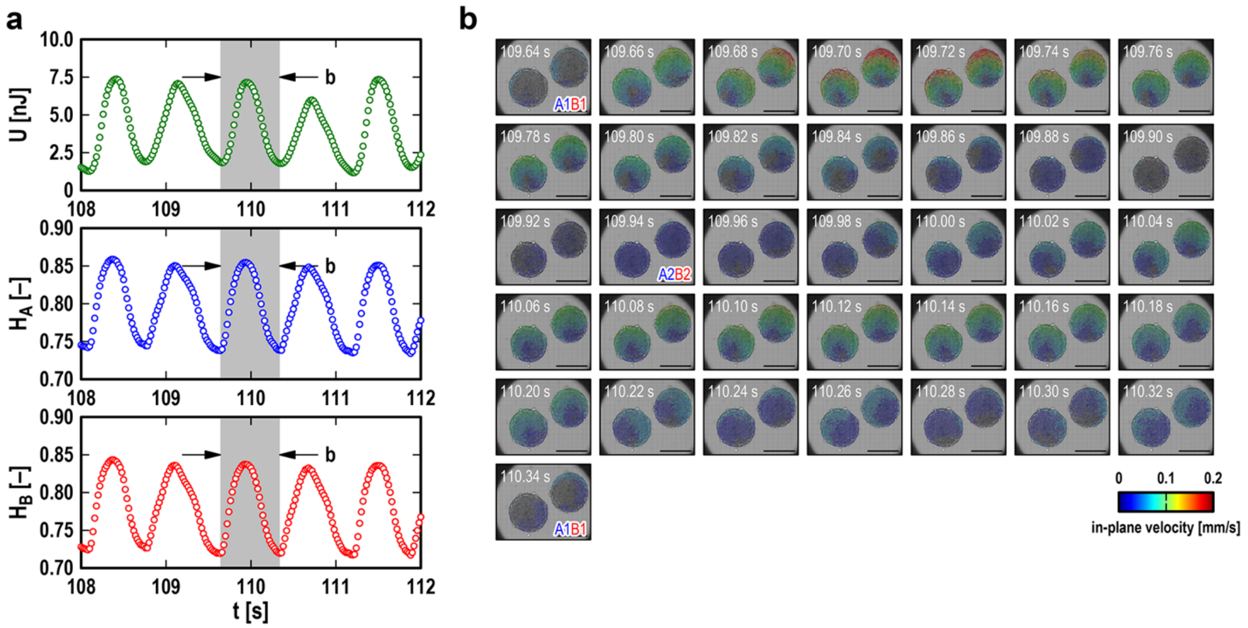

Figure S2 | The regular synchronous beating of a pair of cardiac spheroids (CSs) under compression. a: Temporal changes in the potential energy of the probe, U (top graph), and the normalized heights of the CSs, H_A (middle graph; for the left CS in the snapshots in **b** and **c**) and H_B (bottom graph; for the right CS in the snapshots in **b** and **c**), in the range of t = 108–112 s (from **Figs. 4c** and **4d**). **b**: Snapshots of the CSs in the range of t = 109.64–110.34 s (see the light gray band in **a**). The colors on the CSs represent the local in-plane velocity of the CSs calculated by particle image velocimetry. A1 and B1 represent snapshots for the local minima of H_A and H_B, respectively, and A2 and B2 represent the local maxima of H_A and H_B, respectively. The scale bar in each snapshot represents 0.3 mm.
